# Temperature dependence of sensitized Er^3+^ luminescence in silicon-rich oxynitride films

**DOI:** 10.1186/1556-276X-9-489

**Published:** 2014-09-12

**Authors:** Lingbo Xu, Si Li, Lu Jin, Dongsheng Li, Deren Yang

**Affiliations:** 1 State Key Laboratory of Silicon Materials and Department of Materials Science and Engineering, Zhejiang University, Hangzhou 310027, People’s Republic of China

**Keywords:** Erbium, Silicon-rich oxynitride, Luminescence, Temperature dependence, Energy transfer

## Abstract

The temperature dependence of sensitized Er^3+^ emission via localized states and silicon nanoclusters has been studied to get an insight into the excitation and de-excitation processes in silicon-rich oxynitride films. The thermal quenching of Er^3+^ luminescence is elucidated by terms of decay time and effective excitation cross section. The temperature quenching of Er^3+^ decay time demonstrates the presence of non-radiative trap states, whose density and energy gap between Er^3+^^4^*I*_13/2_ excited levels are reduced by high-temperature annealing. The effective excitation cross section initially increases and eventually decreases with temperature, indicating that the energy transfer process is phonon assisted in both samples.

## Background

Incorporating rare earth (RE) ions into semiconductors and glasses has aroused much research interest in the last decades in view of potential optoelectronic applications [1]. The shielded 4*f* levels of RE^3+^ ions would give rise to optical and electronic phenomena that are almost independent of the host matrix. Among RE ions, Er^3+^ is of particular interest due to the ^4^*I*_13/2_ → ^4^*I*_15/2_ transition at 1.54 μm, which corresponds to the minimum attenuation in commonly used silica optical fibers. Silicon has been one of the most studied hosts for erbium with the aim of photonic-electronic integration. Erbium in silicon can be efficiently excited through electron–hole pair recombination or by impact of energetic carriers [2,3]. However, luminescence of Er^3+^ ions in silicon undergoes a significant thermal quenching due to Auger de-excitation and energy back transfer processes [2,4]. To address such challenge, researchers have tried to embed Er^3+^ ions in hosts with larger bandgap [5], such as silicon-rich oxide (SRO) or silicon-rich nitride (SRN) [6-15]. Recently, silicon-rich oxynitride (SRON) materials have been studied as optical platforms for erbium doping [16-19]. Er-doped SRON (Er:SRON) shows intense 1.54-μm photoluminescence (PL), and non-resonant Er excitation by energy transfer from localized states and silicon nanoclusters (Si-NCs) has been demonstrated [17,19]. In addition, due to the flexibly tunable band structure [20,21], efficient and equivalent carrier injections can be acquired in SRON [22], which acts as a promising host matrix of Er^3+^ ions for electrically pumped light-emitting devices. Cueff et al. [14] have systematically compared the electroluminescence (EL) properties of erbium in SiO_2_, Si_3_N_4_, and SiN_*x*_, and they found that insertion of nitrogen would help enhance electrical conduction at the cost of losses in the external quantum efficiency. The excitation of Er in silicon nitride-based devices is via energy transfer from sensitizers as demonstrated by Yerci et al. [23], and the degraded EL efficiency can be ascribed to the unbalanced carrier injection in silicon nitride. To make a balance between the onset voltage and the EL efficiency, it is helpful to use SRON as the hosts for Er doping. Up to now, the temperature dependence of energy transfer mechanisms in Er:SRON has not been investigated and deserves intensive investigation to engineer efficient light-emitting devices based on Er:SRON.

In this paper, we study the temperature-dependent PL measurements performed on Er:SRON films from 20 to 300 K. Two sets of samples annealed at 600°C and 1,100°C, typically without and with Si-NCs, are chosen. The thermal quenching of Er^3+^ PL intensity is elucidated by studying the temperature dependence of Er^3+^ decay time and effective excitation cross section.

## Methods

Er:SRON films containing 18 at.% excess Si and 0.4 at.% Er were deposited onto the Si (100) substrates by reactive co-sputtering of Er, Si, and Si_3_N_4_ targets in Ar-diluted 1% O_2_ atmosphere. After deposition, the films were annealed for 1 h under N_2_ flux at 600°C or 1,100°C. Transmission electron microscopy measurements showed the absence of Si aggregates in the 600°C annealed sample, while Si-NCs could be clearly observed in the 1,100°C annealed sample [19]. An Edinburgh Instruments FLS-920 fluorescence spectrophotometer (Edinburgh Instruments, Livingston, UK) was employed, with a xenon lamp as the excitation source in the steady-state PL measurements. A microsecond lamp and a picosecond laser diode were used in the transient PL measurements. The samples were put on the cold finger of a closed-loop He cryostat and kept in a vacuum during the low-temperature measurements. The matrix-related PL spectra were corrected for the system spectra response. A more detailed description of the experimental procedures can be found in [19].

## Results and discussion

**Figure 1:**
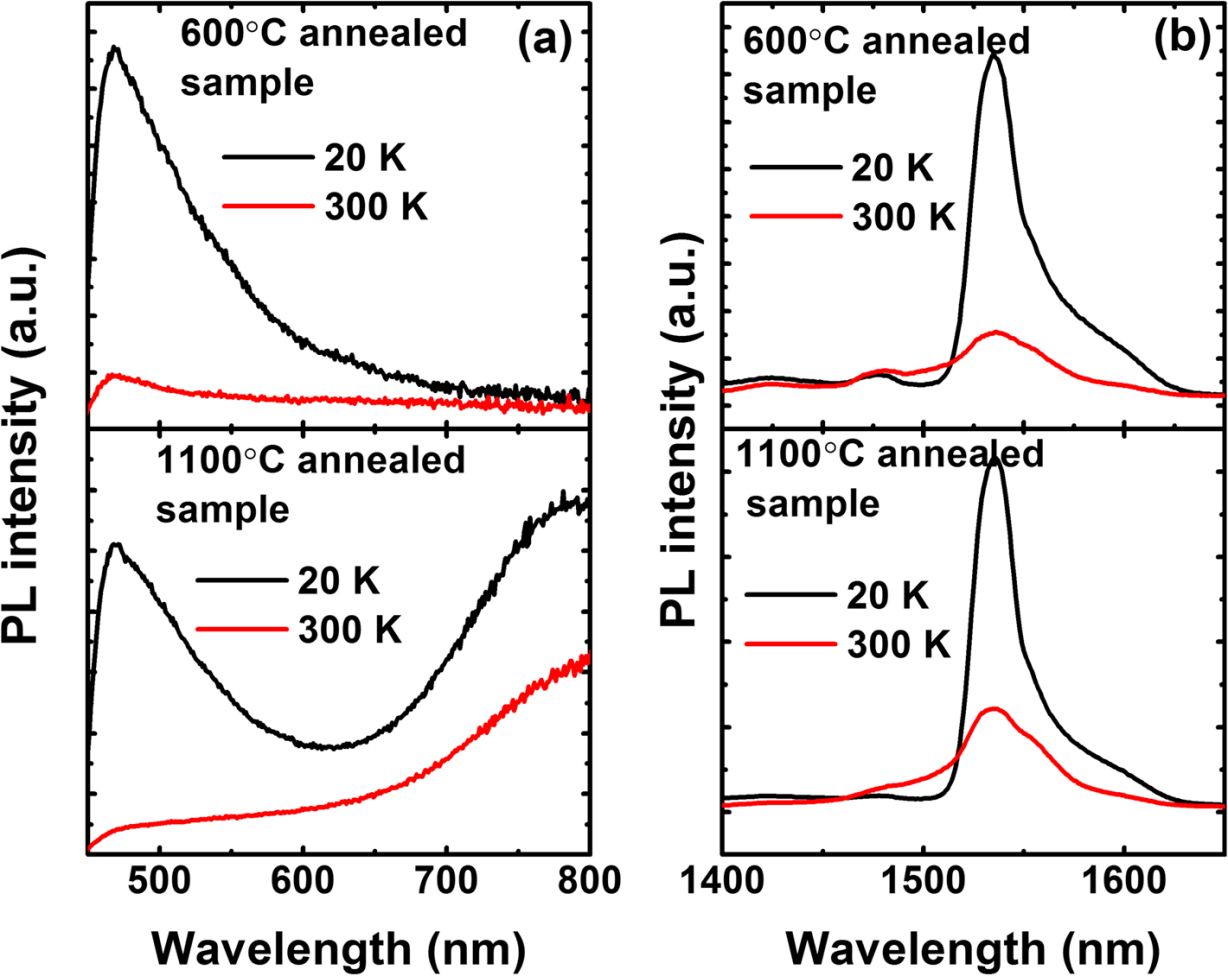
**PL spectra measured at 20 and 300 K. (a)** Matrix-related PL spectra in the visible range and **(b)** Er-related PL spectra in the infrared range for 600°C and 1,100°C annealed samples measured at 20 and 300 K.

Figure 1 shows the PL spectra of the investigated samples measured at 20 and 300 K. Both Er- and Si-NC-related PL bands are observed simultaneously in the 1,100°C annealed sample, while the Si-NC-related PL band is absent in the 600°C annealed sample. At low temperatures, a PL band centered at approximately 468 nm can be clearly observed in both samples. This band undergoes a significant thermal quenching upon heating, being nearly indistinguishable at 300 K. PL decay measurements show that this band has a characteristic lifetime of about 5 ns, and thus, it is attributed to defect-related luminescence [24,25]. We note that the Er-related emission broadens at room temperature, with more contributions from the higher energy side of the spectra. Indeed, the population redistribution in the crystal-field-split manifold of ^4^*I*_13/2_ and ^4^*I*_15/2_ sublevels by thermalization gives rise to the observed broadening. Additionally, thermal quenching of Er- and matrix-related PL, commonly ascribed to a competing non-radiative channel on the sensitizers, is observed in both samples. However, the sensitization mechanisms of Er^3+^ ions must be clarified before the discussion of thermal dynamics.

**Figure 2:**
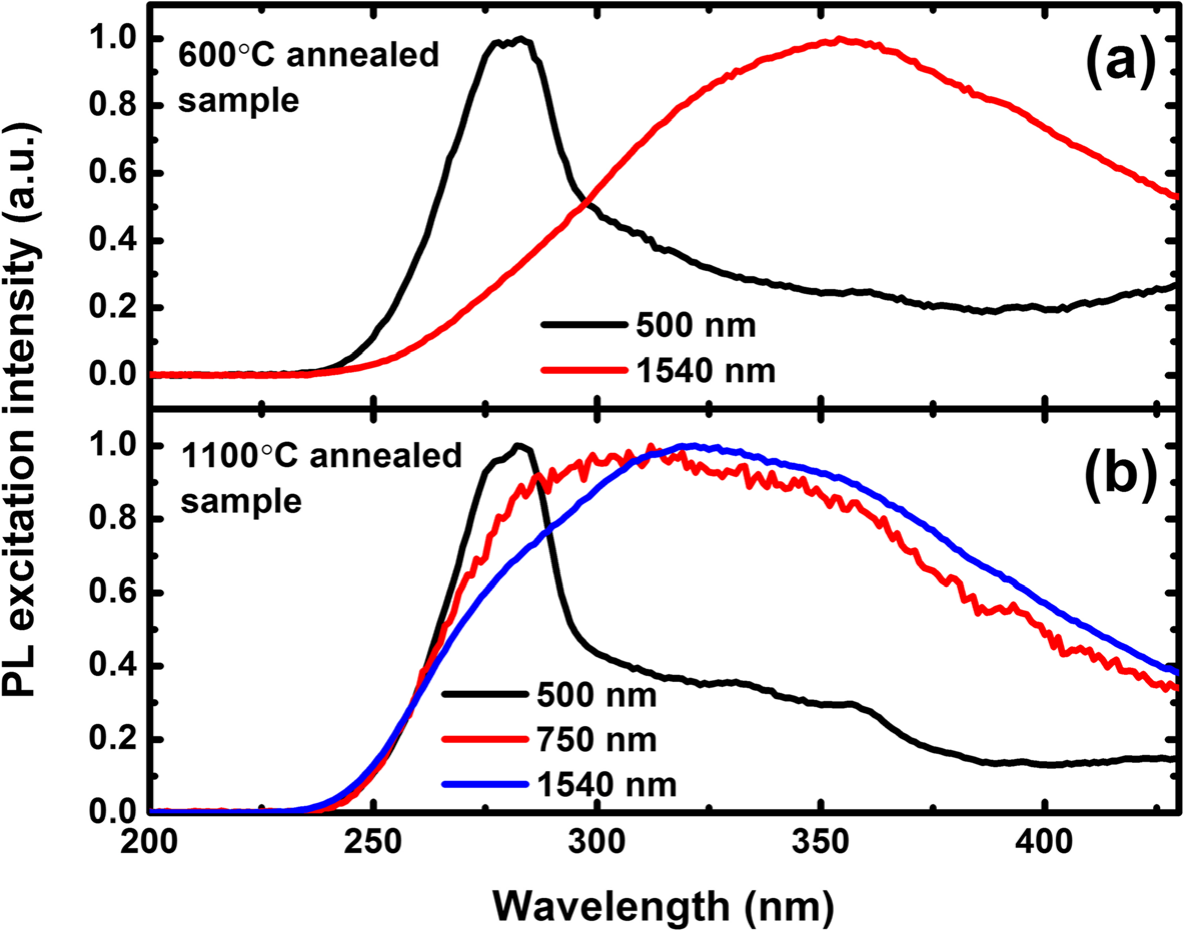
**Normalized PL excitation spectra. (a)** Normalized PL excitation spectra for PL at 500 and 1,540 nm in the 600°C annealed sample. **(b)** Normalized PL excitation spectra for PL at 500, 750, and 1,540 nm in the 1,100°C annealed sample.

Figure 2 shows the normalized PL excitation (PLE) spectra of samples measured at 20 K. PL at wavelengths of 500, 750, and 1,540 nm, typically from defects, Si-NCs, and Er^3+^ ions, has been chosen to identify the relationship between their luminescence. The broad PLE bands of Er^3+^ ions, with no direct Er^3+^ absorption peaks superimposed on, clearly demonstrate the non-resonant excitation of Er^3+^ in all the samples. At a glance, we note that the excitation spectra of defect-related PL show an absolutely different wavelength dependence from those of Er-related PL. Contrarily, the PLE spectra of Si-NCs show a similar shape to those of Er^3+^ ions. The abovementioned PLE characterization result indicates that the defect-related levels emitting at *λ* = 500 nm are not involved in energy transfer processes, while Si-NCs act as sensitizers for Er^3+^ ions in the 1,100°C annealed sample. Moreover, this result suggests that the sensitizers for Er^3+^ ions in the 600°C annealed sample are totally dark. We believe that the Er^3+^ ions in the 600°C annealed sample are mainly sensitized by the localized states in SRON [19]. The 325-nm excitation peak for PL in the 1,100°C annealed sample is indeed a compromise between the excitation and de-excitation processes of carriers in Si-NCs. The absorption cross section of Si-NCs increases with the energy of incident photons while the radiative recombination probability decreases at the same time, since energetic carriers in Si-NCs suffer from thermalization into efficient non-radiative trap states [13].

**Figure 3:**
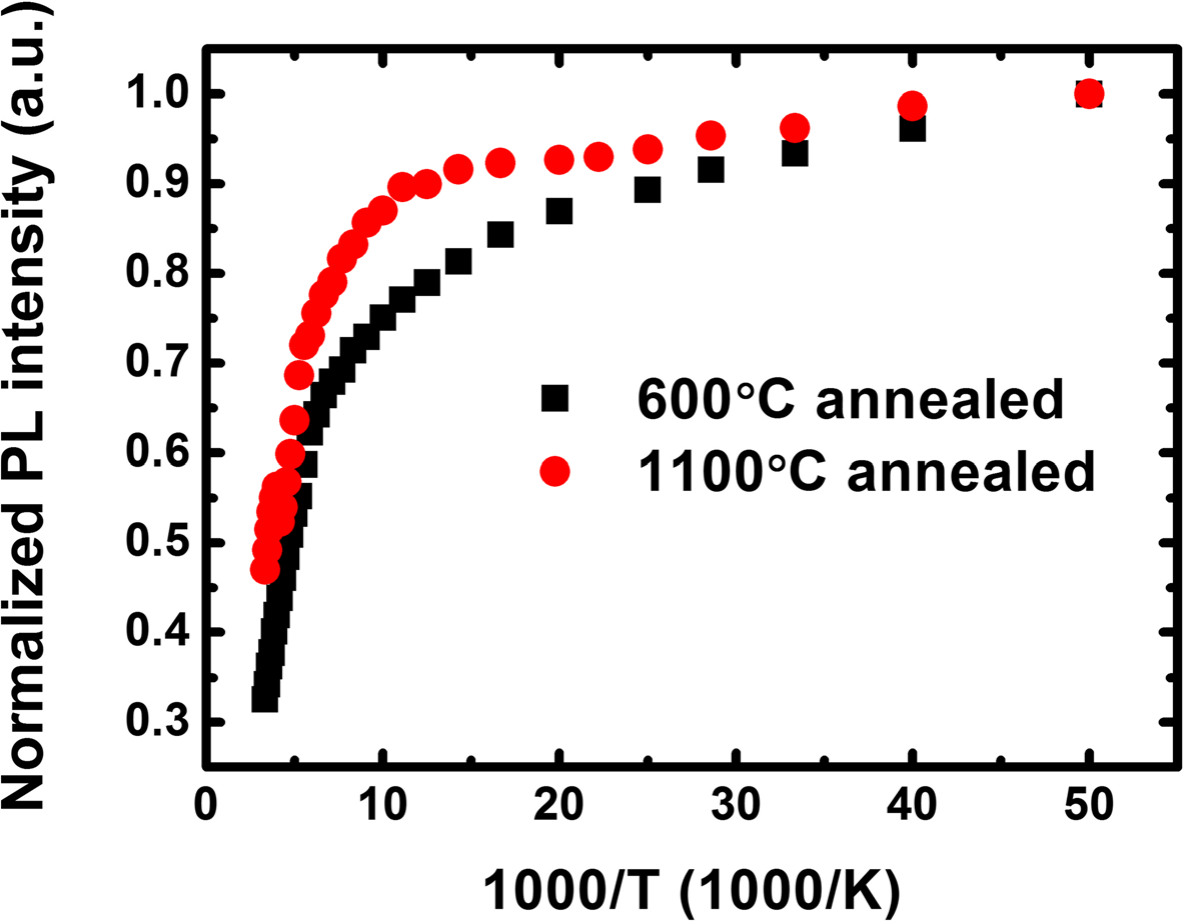
**Emission thermal quenching of Er**^**3+**^**.** Normalized PL intensity of Er^3+^ ions as a function of temperature for the 600°C and 1,100°C annealed samples in an Arrhenius plot.

Figure 3 shows the temperature dependence of integrated Er-related PL intensity (*I*_PL_) for samples annealed at 600°C and 1,100°C in an Arrhenius plot. The PL intensity of the samples is normalized to their values at 20 K. *I*_PL_ drops from 20 K to room temperature by a factor of 3 and 2 for the 600°C and 1,100°C annealed samples, respectively. The thermal quenching of Er^3+^ luminescence in SRON is rather small and is comparable to that in SRO [8] as well as in SRN [13]. We try to fit the curves with an Arrhenius equation, yet we find that the fittings do not converge. This indicates that the thermal quenching of sensitized Er^3+^ PL in SRON is different from that in SRO and cannot be solely explained by thermal emission of the carriers out of a confining potential [11]. In the linear excitation regime, we have [26]:

(1) $$I_{\mathrm{PL}}\sim \phi \sigma_{\mathrm{Er}}N_{\mathrm{Er},\mathrm{sen}}\frac{\tau_{\mathrm{dec}}}{\tau_{\mathrm{rad}}}$$

where *ϕ* is the photon flux, *σ*_Er_ is the effective excitation cross section of Er^3+^ ions, *N*_Er, sen_ is the density of sensitized Er^3+^ ions, *τ*_dec_ is the decay time, and *τ*_rad_ is the radiative lifetime. Assuming that *N*_Er, sen_ and *τ*_rad_ are temperature independent, the reason for the thermal quenching of *I*_PL_ will be elucidated in terms of *τ*_dec_ and *σ*_Er_.

**Figure 4:**
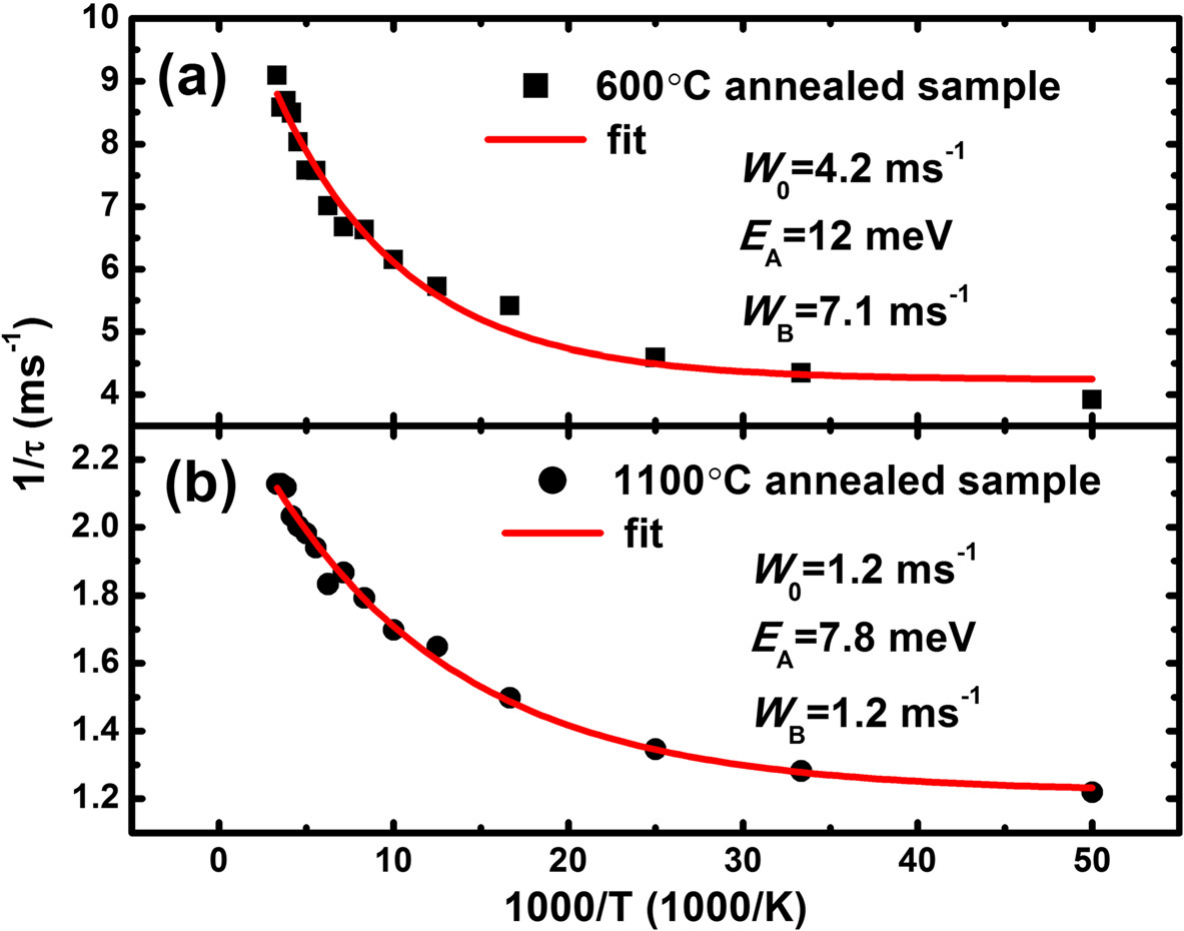
**Temperature dependence of 1/*τ***_**dec**_**.** Temperature dependence of 1/*τ*_dec_ for the 600°C **(a)** and 1,100°C **(b)** annealed samples in an Arrhenius plot.

Figure 4 shows the temperature dependence of 1/*τ*_dec_ for our samples. The samples annealed at 600°C and 1,100°C have *τ*_dec_ of 0.25 and 0.82 ms at 20 K, respectively. These quantities decrease to 0.11 and 0.47 ms at room temperature, indicating the existence of non-radiative trap states which interact with excited Er^3+^ ions. The decay time data can be modeled as follows [13]:

(2) $$1/\tau_{\mathrm{dec}}=W_{0}+W_{B}\exp\left(-E_{A}/\mathrm{kT}\right)$$

where *W*_0_ is the decay rate at *T* = 0, *E*_A_ is the activation energy of non-radiative trap states, and *W*_B_ is the rate constant of the trap. By fitting the decay data with this model, we obtain that *W*_0_ is 4.2 and 1.2 ms^−1^, *E*_A_ is 12 and 7.8 meV, and *W*_B_ is 7.1 and 1.2 ms^−1^ in the 600°C and 1,100°C annealed samples, respectively. The value of *W*_0_ for the 600°C annealed sample is larger than that for the 1,100°C annealed sample. This is due to the change in the Er^3+^ environment and in the interaction of Er^3+^ with the local field of the embedding medium [27], as it is expected that the 600°C annealed sample is homogeneously amorphous while the 1,100°C annealed sample is phase separated. The decrease of *E*_A_ with increasing annealing temperature indicates the narrowing of energy gap between the Er^3+^^4^*I*_13/2_ excited level and the trap states, and the decrease of *W*_B_ is ascribed to the gradual passivation of non-radiative trap states during thermal treatments.

After a simple transformation of formula (1), we have formula (3) as follows:

(3) $$\sigma_{\mathrm{Er}}\sim \frac{\tau_{\mathrm{rad}}}{\phi N_{\mathrm{Er},\mathrm{sen}}}\cdot \frac{I_{\mathrm{PL}}}{\tau_{\mathrm{dec}}}$$

**Figure 5:**
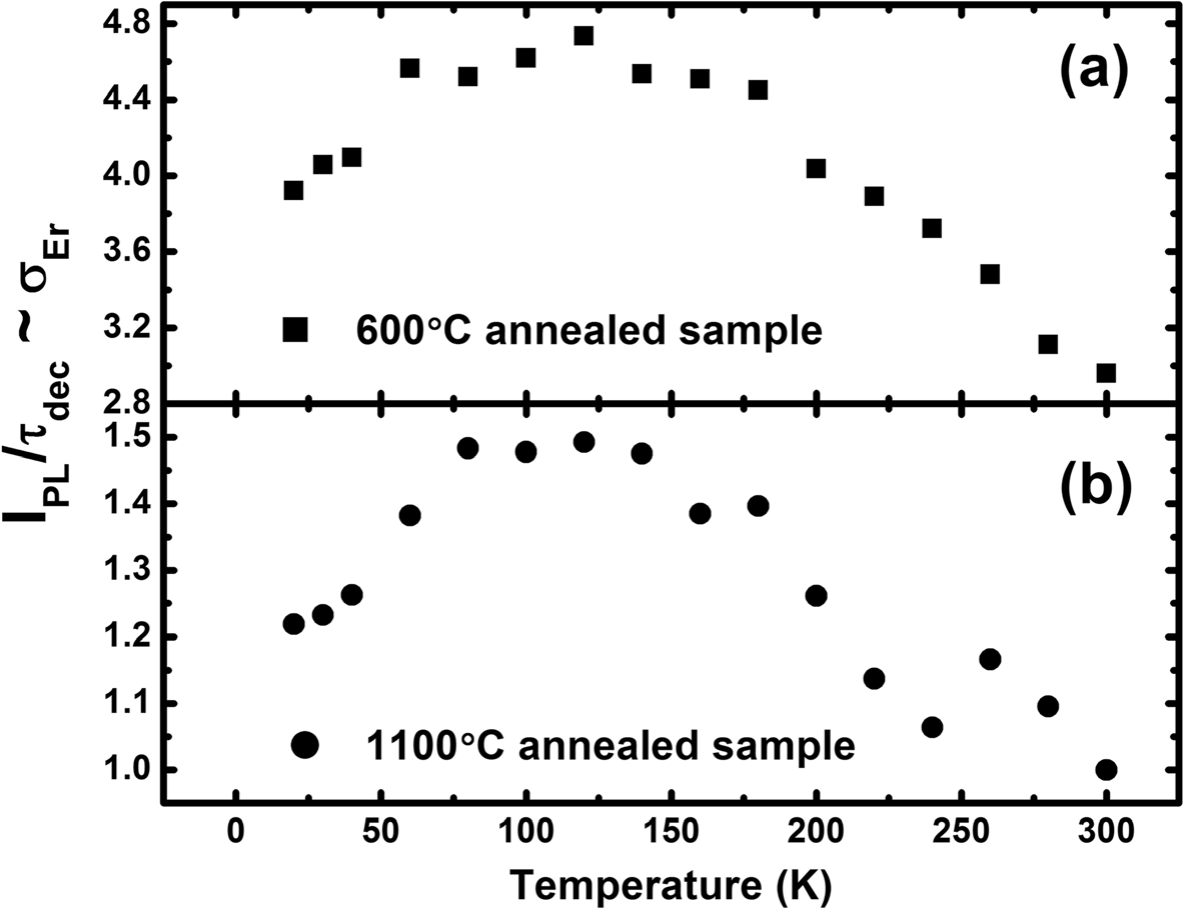
**Temperature dependence of *σ***_**Er**_**.** Temperature dependence of *σ*_Er_ for the 600°C **(a)** and 1,100°C **(b)** annealed samples.

Thus, the ratio *I*_PL_/*τ*_dec_ gives information on *σ*_Er_ in relative units, as shown in Figure 5. The *σ*_Er_ for the 600°C and 1,100°C annealed samples is found to initially increase with temperature until 140 K before it starts to decrease at higher temperatures. This is similar to the case in Er-doped SRN [13] and implies that the energy transfer process is phonon assisted in both samples, regardless of the sensitization mechanism. For the 1,100°C annealed sample in which the sensitization of Er^3+^ ions is via Si-NCs, by emitting or absorbing phonons, the momentum conservation rule of electron–hole recombination in Si-NCs is satisfied and the energy mismatch between the recombination energy of excitons and the Er^3+^ 4*f*-shell energy is bridged [28,29]. For the 600°C annealed sample in which the sensitization of Er^3+^ ions is via localized states, the localization of an exciton in a small space partially breaks the momentum conservation rule and makes the direct recombination of the exciton possible. However, the energy mismatch may be large, and the increasing number of phonons with temperature is beneficial to bridge it, leading to the increase in energy transfer rate. The temperature dependence of *σ*_Er_ is thus dominated by the competition between the increasing energy transfer rate and the decreasing excited state density of sensitizers with temperature [13]. Generally, the non-radiative channels are gradually activated with increase in temperature, and their coupling with sensitizers is strengthened, leading to the decrease in excited state density of sensitizers as well as *σ*_Er_. It seems that the increase in energy transfer rate is dominant in the low temperature range, while the decrease in the excited state density of sensitizers prevails in the high temperature range.

## Conclusions

In conclusion, we have studied the temperature dependence of sensitized Er^3+^ emission via localized states and Si-NCs in silicon-rich oxynitride films. Lifetime measurements have proved the existence of non-radiative trap states in both samples, whose density and energy gap between Er^3+^^4^*I*_13/2_ excited levels are reduced by high-temperature annealing. Energy transfer from localized states and Si-NCs to Er^3+^ ions is found to be phonon assisted. The thermal dynamics of *σ*_Er_ is thus determined by the increasing energy transfer rate and the decreasing excited state density of sensitizers with temperature. Indeed, the thermal quenching of sensitized Er^3+^ luminescence is small in both samples, indicating that stable room temperature operation of devices based on Er:SRON is possible.

## Abbreviations

Er:SRON: erbium-doped silicon-rich oxynitride; IPL: Er-related photoluminescence intensity; PL: photoluminescence; PLE: photoluminescence excitation; RE: rare earth; Si-NCs: silicon nanoclusters; SRN: silicon-rich nitride; SRO: silicon-rich oxide; SRON: silicon-rich oxynitride.

## Competing interests

The authors declare that they have no competing interests.

## Authors’ contributions

LX carried out the experiments, analyzed the data, and drafted the manuscript; SL and LJ contributed to the analysis of the results in this paper; DL and DY conceived the study, participated in the result discussion, and revised the manuscript. All authors read and approved the final manuscript.
